# Eulenberg

**Published:** 1884-01

**Authors:** 


EULENBERG:—The examination of hogs for trichinæ and cysticerci (measles) in Prussia: during the year 1882.—*Veirteljahrsschrift f. gericht medicin.* Berlin, Oct., 1883.

| | |
|---|---|
| Whole number hogs examined | 3,808.142 |
| Trichinous | 1,852 |
| Number of pieces of American pork found trichinous | 1,365 |
| Number of swine having 'measles' | 13,564 |
| Number of pork examined | 20,140 |

The importation of American pork seems to have decreased before the government placed restrictions, or an entire embargo upon it. In Minden 51,427 pieces were imported in 1881, while in 1882 only 30,000: at Wiedenbrück in 1881, 21,925 pieces were examined while in 1882 only 4,608 were imported.

Sixty soldiers of one regiment, became very ill from eating some (German) pork in a raw state—hams, smoked sides, etc., which a butcher had stolen and sold to them. B.

THE GREATEST ADDITION to veterinary literature which has been made during the past ten year is the "Zeitschrift für Vergleichende Augenheilkunde, edited by Prof. Dr. Berlin of Stuttgart and Dr. Eversbusch of Munich. The issue for 1883, comes out in one number a supplement to the "Deutshe Zeitshrift für Thiermedicine, published by Vogel in Leipzig.

From the former we take the following "notes":

